# Skin Lipids and Their Influence on Skin Microbiome and Skin Care

**DOI:** 10.1021/acsomega.4c11687

**Published:** 2025-07-02

**Authors:** Raquel Allen Garcia Barbeto Siqueira, Iveta Hradkova, Vânia Rodrigues Leite-Silva, Newton Andréo-Filho, Patricia Santos Lopes

**Affiliations:** † Programa de Pós-Graduação em Medicina Translacional, Departamento de Medicina, Escola Paulista de Medicina, 28105 Universidade Federal de São Paulo, São Paulo 04021-001, Brazil; ‡ Department of Dairy, Fat and Cosmetics, 52735 University of Chemistry and Technology Prague, Prague 166 28, Czech Republic; § Therapeutics Research Centre, The University of Queensland Diamantina Institute, Translational Research Institute, Brisbane, Queensland 4102, Australia; ∥ Departamento de Ciências Farmacêuticas, 505146 Instituto de Ciências Ambientais, Químicas e Farmacêuticas, Universidade Federal de São Paulo, UNIFESP, Diadema 09913-030, Brazil

## Abstract

Skin lipids are essential components that play crucial roles in maintaining the skin barrier, preventing transepidermal water loss, and protecting against external agents. The specific composition of lipids may vary according to factors, such as age, diet, and environmental conditions. They are found predominantly in the stratum corneum, the outermost layer of the epidermis, with lipids originating from epidermal lipids and also from the sebaceous glands. The diversity of lipids, including ceramides, cholesterol, free fatty acids, sphingolipids, phospholipids, triglycerides, and waxes, reflects the complexity of their functions. Understanding the properties and biosynthesis of skin lipids is fundamental for advancing dermatology, developing treatments for various skin conditions, and maintaining the integrity of the skin barrier. Skin microbiome could affect skin lipid composition, and this topic has yet to be completely understood. This literature review aims to understand the properties of lipids found in the skin, the function and importance of fatty acids for skin maintenance and integrity, and their correlations that influence homeostasis: pH, the role of lipids in the microbiota, and finally, daily care practices that can influence the health of the skin and also the microbiome.

## Graphical abstract

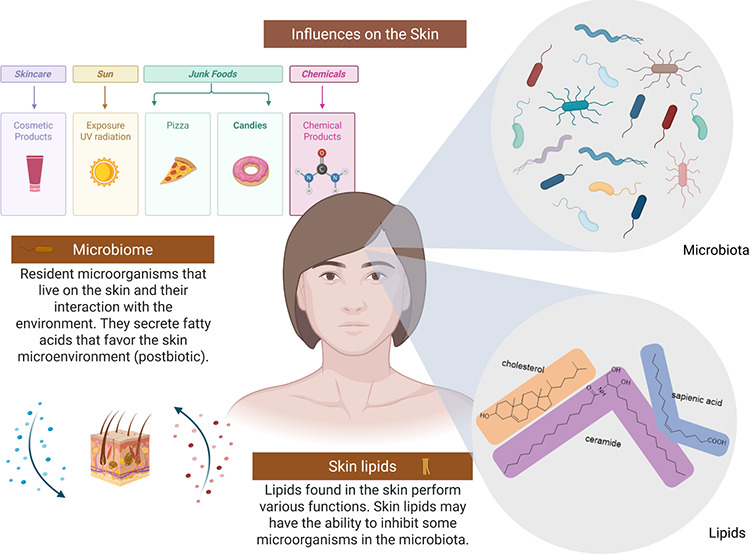

## 1 Introduction

The skin is one of the most important organs in the body, as it delimits what is inside and what is outside, serving as an important protective barrier and an indispensable component of innate immunity. It is divided into three layers: epidermis, dermis, and hypodermis. A recent study conducted by Rajkumar et al. classified the skin as a barrier composed of four layers: physical, chemical, microbiological, and immunological. Together, these layers maintain structural stability and hydration, prevent dysbiosis, and remove cutaneous inflammation.^1^

Epidermis—the most superficial layer of the skin—present four sublayers with specific functions and structures: *stratum basale* (SB)—the deepest layer, *stratum spinosum* (SS), *stratum granulosum* (SG), and *stratum corneum* (SC).^2^ The SC is the most superficial layer of the epidermis, formed by the stacking of approximately 15 to 25 layers of anucleated cells joined by cellular structures called corneodesmosomes. These cells form an arrangement known as “brick and mortar” (Figure 1), as they are immersed in a lipoprotein matrix formed by proteins (75–80%)—the bricks—and lipids (5–15%)—the mortar.^2,3^

**Figure 1:**
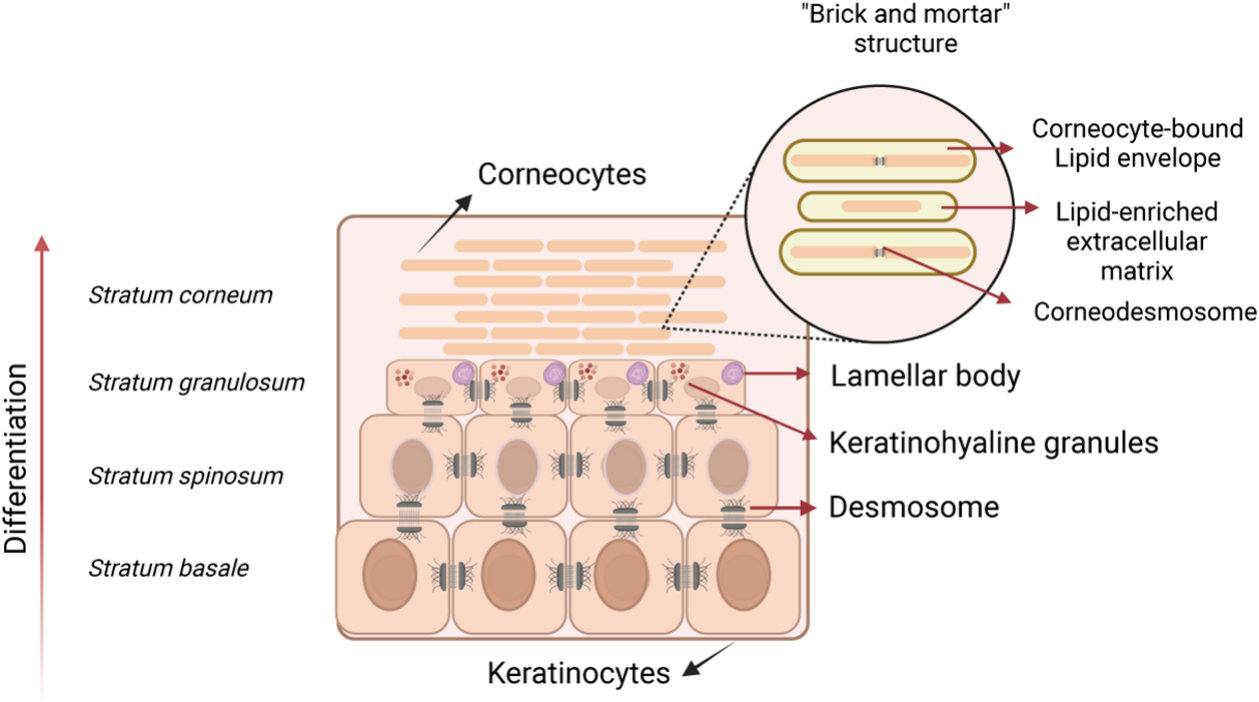
Differentiation of epidermis showing the four sublayers of skin and “brick and mortar” structure. Created with permission from BioRender.com.

## 2 Types of Skin Lipids

Lipids are hydrophobic molecules that are essential to the skin, as they perform numerous important functions, such as inhibiting transepidermal water loss (TEWL) and preventing the entry of microorganisms or other substances by preserving the skin’s barrier function.^2^ Lipid biosynthesis in the skin is regulated by several factors, including hormones, cytokines, environmental factors, aging, and exposure to ultraviolet (UV) rays, which can negatively affect lipid synthesis and compromise the skin’s barrier function.^3^

Lamellar bodies are organelles found in keratinocytes, containing a high concentration of enzymes, along with cholesterol sulfates, phospholipids, glycosylceramides, and sphingomyelins in their composition.^4–5^ The enzymes are responsible for the synthesis and processing of lipids and their delivery into the extracellular environment. In this sense, lipids are synthesized by keratinocytes during epidermal differentiation and are very abundant in the epidermis, forming an envelope around the corneocytes (which are formed from keratinocytes during cornification when water concentration decreases and the cell nucleus disappears); in addition, the extracellular space is rich in different types of lipids, forming a unique lipid matrix.^2^ Among these, the primary lipids present in the stratum corneum (SC)—the outermost layer of the epidermis—include ceramides (50%), cholesterol (25–27%), free fatty acids (10–15%), cholesterol esters (10%), and other less abundant lipids.^2,6,7^

Ceramides, which represent the majority of the lipids in the *stratum corneum,* are particularly important for the cohesion and integrity of the skin barrier.^6,8^ Covalently linked ceramides A and B form a structure to which free ceramides, free fatty acids, and cholesterol are later added in the SC.^3^ Studies have shown that the absence or deficiency of ceramides is associated with several skin diseases, including atopic dermatitis and psoriasis.^4,5^

Phospholipids, although less abundant in the stratum corneum, are important components of cell membranes in the deeper layers of the epidermis. They are precursors of free fatty acids and sphingolipids and play critical roles in cell signaling and in maintaining membrane integrity.^9^ In addition to their functions, skin lipids play critical roles in cell signaling and immune response. They help regulate cell proliferation and differentiation and have antimicrobial properties that protect the skin from infection. Lipids such as free fatty acids (FFA) also have anti-inflammatory properties, contributing to skin homeostasis.^6,7^

## 3 Biosynthesis of Skin Lipids

Ceramides (Cer) are sphingolipids composed of sphingosine and a fatty acid (Figure 2). There are 12 types of ceramides most abundant in the SC derived from glucosylceramide and sphingomyelin, each with specific structures and functions.^4,10^ They are essential for the formation of the lamellar lipid layer, which prevents dehydration and protects against the entry of harmful substances.^10,11^ Cer biosynthesis occurs mainly in keratinocytes, and this process involves several enzymatic steps. The enzyme ceramide synthase plays a crucial role in this process, catalyzing the binding of sphingosine to fatty acids.^11^ In addition to ceramides, the skin contains other sphingolipids, such as sphingomyelin and glucosylceramides.^10^

**Figure 2:**
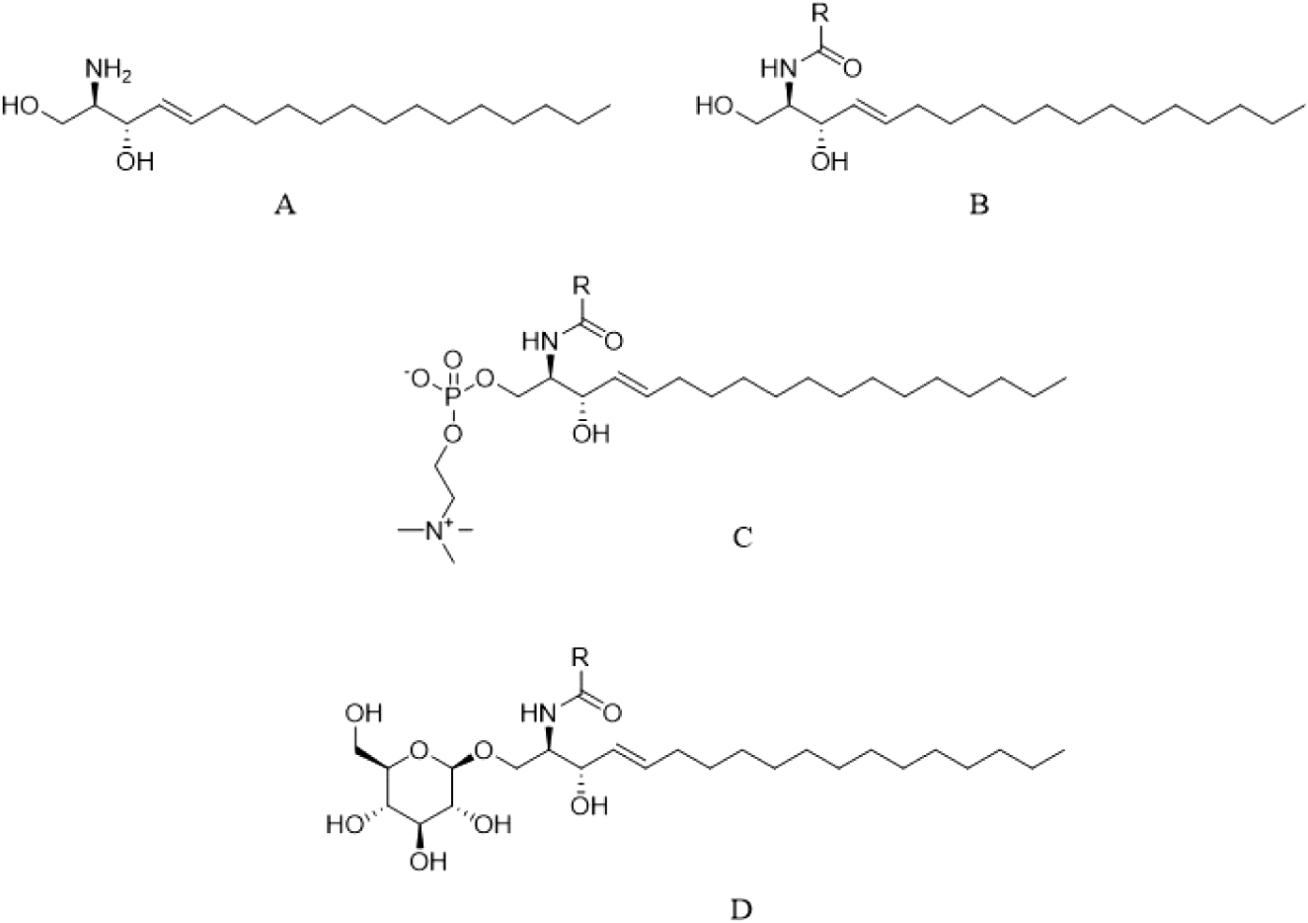
Representation of the main ceramide molecules present in human skin: sphingosine (A), ceramide (B), sphingomyelin (C), and glycosylceramide (D).

Sphingomyelin can be converted into ceramides through the action of the enzyme sphingomyelinase, thus contributing to skin lipid homeostasis.^11^ After the release of lipids and enzymes from the granular bodies, phospholipids and glycosylceramides are converted by hydrolytic enzymes into mature ceramides, which along with fatty acids and cholesterol, form the barrier in the SC.^4^

Figure 3 shows the biosynthesis pathways for ceramide production, starting from palmitoyl-CoA and serine, as well as the biosynthesis of cholesterol and fatty acids, starting from acetyl-CoA. These biochemical reactions rely on enzymes such as serine palmitoyl-transferase, 3-hydroxy-3-methylglutaryl coenzyme A reductase (HMG-CoA), and aAcetyl-CoA carboxylase, which are pH-dependent. The significance of pH is discussed later.

**Figure 3:**
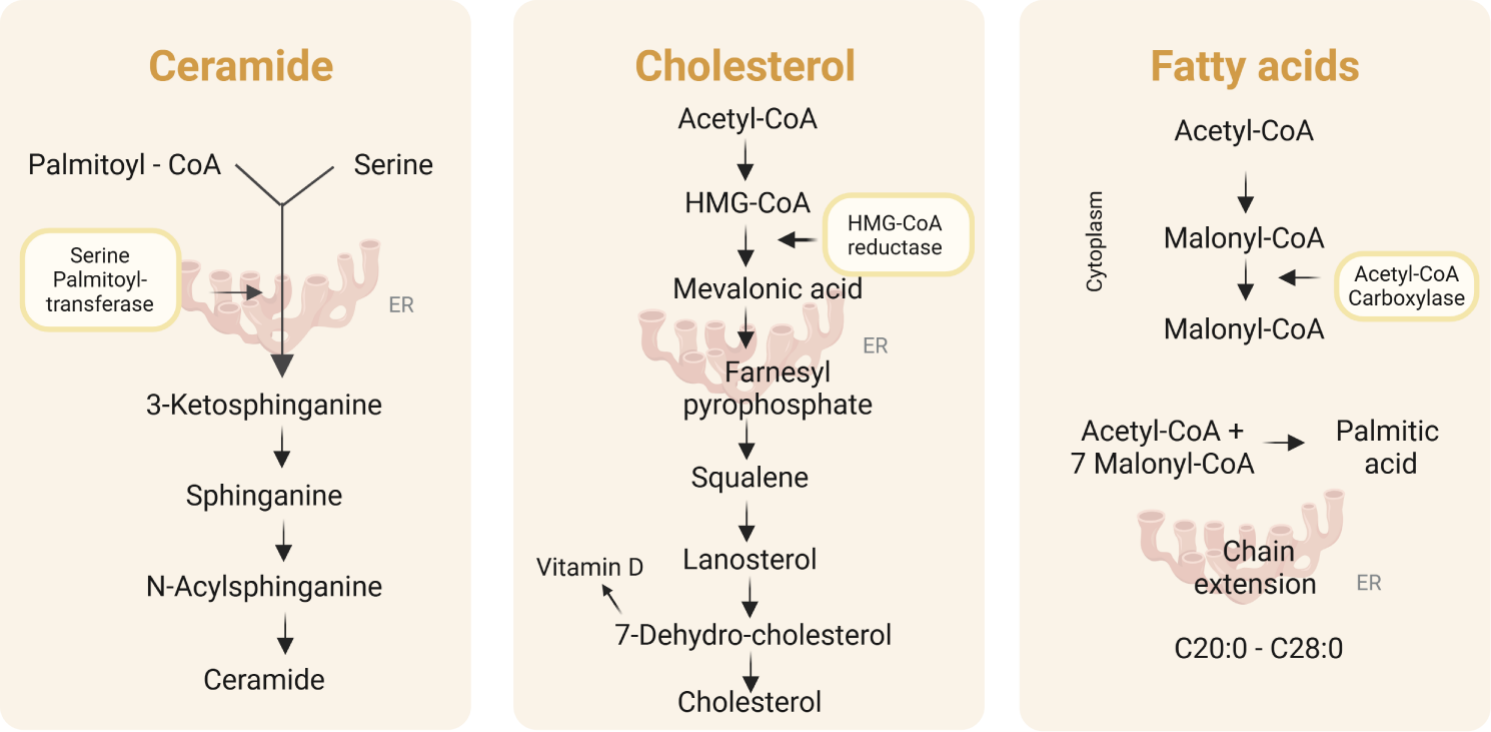
Ceramide, cholesterol, and fatty acid biosynthetic pathways. Created with BioRender.com.

Cholesterol and free fatty acids are other essential components of cutaneous lipids, synthesized in the skin through well-established metabolic pathways. Cholesterol is produced through the mevalonate pathway (Figure 3), while fatty acids are synthesized from acetyl-CoA through the action of fatty acid synthase. These lipids are then transported to the stratum corneum, where they contribute to the formation of lamellar layers.^4,12^ Cholesterol contributes to the fluidity and stability of cell membranes, while free fatty acids have antimicrobial and anti-inflammatory properties. Both lipids are essential for maintaining skin homeostasis and protecting against infections.^6,12^

## 4 Functions of Skin Lipids

Cholesterol is an indispensable component of the skin barrier, representing around 25% of *stratum corneum* lipids. The composition of fatty acids will be discussed further later. Waxes and cholesterol esters are minor but important components of sebum. They help form a hydrophobic layer on the surface of the skin, which contributes to moisture retention and protection against dehydration. Furthermore, these lipids help protect against pathogens.^2,13–16^

Triglycerides are stored in the sebocytes within sebaceous glands and are released onto the surface of the skin as part of sebum. They are important for the lubrication of the skin and hair and are stored in adipocytes as an energy reserve. In addition, sebum also has skin immunomodulatory properties.^2^ The hydrolysis of triglycerides by lipase enzymes releases free fatty acids, which have antimicrobial properties.^14^ Table 1 summarizes the main lipids found in the skin.

**Table 1:** Main Lipids Found in the Skin

Lipids	Function/Activity	References
Ceramides	Barrier function	Elias and Feingold, 2006;^16^ Feingold, 2007;^5^ Hannun and Obeid, 2008; Knox and Boyle, 2021;^2^ Schild et al., 2023;^107^ Yong et al., 2025 ^108^
Cholesterol	Barrier function/plasma membrane	Downing and Stewart, 2000;^12^ Elias and Feingold, 2006;^16^ Feingold, 2007;^5^ Knox and Boyle, 2021 ^2^
Waxes	Barrier function	Schurer and Elias, 1991;^13^ Elias and Feingold, 2006;^16^ Knox and Boyle, 2021 ^2^
Fatty acids	Antimicrobial and anti-inflammatory	Elias and Feingold, 2006;^16^ Feingold, 2007;^5^ Knox and Boyle, 2021 ^2^
Sphingolipids	Barrier function/cell signaling	Elias and Feingold, 2006;^16^ Hannun and Obeid, 2008;^109^ Knox and Boyle, 2021 ^2^
Phospholipids	Plasma membrane/signaling	Elias and Feingold, 2006;^16^ Bouwstra and Ponec, 2006;^9^ Knox and Boyle, 2021 ^2^
Triglycerides	Sebum production, energy reserve	Elias and Feingold, 2006;^16^ Zouboulis, 2009;^14^ Knox and Boyle, 2021 ^2^

Fatty acids (FAs) are fundamental components of the skin, playing essential roles in maintaining the integrity of the skin barrier, protecting against infections, and regulating inflammation. They are found in various forms in the skin, including free fatty acids, components of ceramides, triglycerides, and phospholipids. Free fatty acids constitute approximately 10–15% of the lipids in the stratum corneum derived from sebaceous lipids, triglycerides broken down by the action of lipase enzymes, and also epidermal lipids from the intercellular environment.^15^

FAs are chemical chains composed of carbon, hydrogen, and oxygen, with a hydrocarbon at one end and a carboxyl group at the other, with a length that can vary from 4 to 36 carbons (C4–C36)^16^. The composition and balance of fatty acids in the skin are crucial to skin health. Deficiencies or imbalances can lead to several dermatological conditions, such as atopic dermatitis, psoriasis, and acne.^2,15^ Studies suggest that supplementation with essential fatty acids can improve barrier function and reduce inflammation, highlighting the importance of fatty acids in maintaining skin health.^17^ Due to these characteristics, FAs are indispensable materials in cosmetic products and can act as emulsifiers, softeners, cleansers, whiteners, etc.^15^

Saturated fatty acids do not have double bonds between carbon atoms, such as palmitic acid (C16:0) and stearic acid (C18:0), which are important components of the skin barrier. They are found predominantly in ceramides and triglycerides in the skin. Saturated fatty acids are known for their moisturizing and protective properties, helping to maintain the structural integrity of cell membranes and prevent transepidermal water loss.^18^

Unsaturated fatty acids, including oleic acid, linoleic acid, alpha-linolenic acid, and sapienic acid (Figure 4), play critical roles in skin barrier function and inflammation regulation. These fatty acids contain one or more double bonds and are subdivided into monounsaturated and polyunsaturated. Linoleic acid is an essential component of ceramides, contributing to the formation of the lipid lamellae that maintain cellular cohesion in the stratum corneum.^19^ A deficiency in linoleic acid can lead to conditions such as atopic dermatitis.^20^

**Figure 4:**
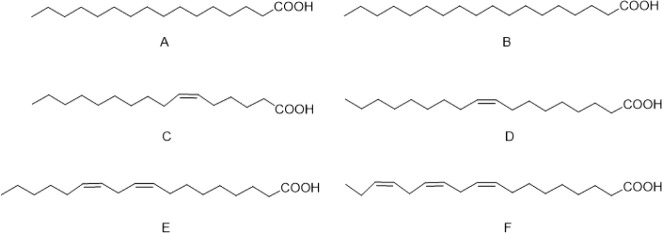
Representation of main fatty acids present in human skin: palmitic acid (A), stearic acid (B), sapienic acid (C), oleic acid (D), linoleic acid (E), and alpha-linolenic acid (F).

Oleic acid, a monounsaturated fatty acid, is abundant in the sebum and intercellular lipids of the stratum corneum. While it has moisturizing properties, an excess can disrupt ceramide organization in the skin barrier, resulting in increased permeability and susceptibility to irritation. Therefore, maintaining the proper balance of oleic acid is crucial for skin health.^12,21^

Linoleic acid, a polyunsaturated fatty acid, is essential for skin barrier function and must be obtained through the diet, as the human body cannot synthesize it.^19^ It is incorporated into ceramides, where it plays a vital role in forming the lipid barrier and maintaining skin hydration. A deficiency in linoleic acid can lead to dry, flaky skin.^22^ Linoleic acid present in ceramides is particularly effective at maintaining cellular cohesion and preventing transepidermal water loss. These interactions are fundamental to the integrity and function of the skin barrier.^19,23^ Alpha-linolenic acid is another essential polyunsaturated fatty acid found in the skin. It has anti-inflammatory properties and can be converted into other long-chain polyunsaturated fatty acids, such as eicosapentaenoic acid (EPA) and docosahexaenoic acid (DHA), which also play anti-inflammatory roles and help maintain the skin barrier.^24,25^

Sapienic acid ((*Z*)-hexadec-6-enoic acid) is the predominant fatty acid in human sebum.^28^ This fatty acid is synthesized by the enzyme Δ-6 desaturase from palmitic acid. It can be converted by elongation and desaturation to sebaleic acid ((5*Z*,8*Z*)-octadeca-5,8-dienoic acid), which is another unique fatty acid found in the sebum. Sapienic acid occurs only in human skin among hair-bearing animals.^26,27^ Lower activity of Δ-6 desaturase and the associated higher ratio of palmitic/sapienic acid (C16:0/C16:1) can play an important role in the development of acne.^28^ Sapienic acid is a potent antimicrobial agent against *Fusobacterium nucleatum*, *Streptococcus sanguinis* *, and* *Streptococcus mitis*.^29^ Neumann et al. determined its strong antistaphylococcal effect and its ability to inhibit determinant virulence production.^30^ When the effect of sapienic acid is compared between *Staphylococcus aureus* and *Staphylococcus epidermidis*, *S. epidermidis* exhibits greater resistance.^31^

Fatty acids can have different chain lengths, which influence different regulatory roles in the skin. Long-chain fatty acids, such as arachidonic acid, are important precursors of eicosanoids, which are signaling molecules involved in the inflammatory response.^32^ Arachidonic acid is released from cell membranes in response to inflammatory stimuli and is metabolized into prostaglandins and leukotrienes, which play roles in inflammatory and immunological processes in the skin.^33,34^ Long-chain fatty acids can also help maintain skin hydration, reduce moisture loss, and resist the entry of harmful agents into the skin, thus maintaining the preserved barrier function. In addition, different FAs can activate or inhibit the immune response.^15^

Short- and medium-chain fatty acids, such as lauric acid, are also found in the skin, especially in the sebum. They possess antimicrobial properties and help protect the skin against bacterial and fungal infections.^35^ Lauric acid has been shown to be effective against several strains of bacteria and is an important component of the skin’s natural defense.^35,36^ Short-chain fatty acids are produced by the metabolism of the resident microbiota and play an important role in inhibiting the growth of pathogenic microorganisms while maintaining the balance of the skin microbiota.^15^ Table 2 summarizes the main fatty acids found in the skin: saturated, monounsaturated fatty acids (MUFA), and polyunsaturated fatty acids (PUFA).

**Table 2:** Mainly Fatty Acids Found in the Skin

Fatty acids	Structure/Saturation	Function/Activity	Reference(s)
Lauric acid	(C12:0) Saturated	Barrier function/antimicrobial activity	Kabara, 1972;^36^ Knox and Boyle, 2021 ^2^
Dodecanoic acid	(C12:0) Saturated	Antimicrobial properties against *C. acnes*	Nakatsuji, 2009;^110^ Knox and Boyle, 2021 ^2^
Myristic acid	(C14:0) Saturated	Regulating inflammation	Knox and Boyle, 2021;^2^ Alonso-Castro, 2022 ^111^
Palmitic acid	(C16:0) Saturated	Barrier funtion	Rawlings and Harding, 2004;^18^ Knox and Boyle, 2021 ^2^
Stearic acid	(C18:0) Saturated	Barrier function	Rawlings and Harding, 2004;^18^ Knox and Boyle, 2021 ^2^
Eicosanoic (arachidic) acid	(C20:0) Saturated	Proinflammatory properties	Knox and Boyle, 2021 ^2^
Behenic acid	(C22:0) Saturated	Emollient	Knox and Boyle, 2021;^2^ Banov, 2014 ^112^
Lignoceric acid	(C24:0) Saturated	Barrier function	Knox and Boyle, 2021;^2^ Stahlberg, 2015 ^113^
Hexacosanoic acid	(C26:0) Saturated	Barrier function	Knox and Boyle, 2021;^2^ Tsuji, 1985 ^114^
Myristoleic acid	(C14:1, *n*-5) MUFA	Antimicrobial properties against *C. acnes*	Kim, 2021;^115^ Knox and Boyle, 2021 ^2^
Palmitoleic acid	(C16:1, *n*-7) MUFA	Barrier function	Knox and Boyle, 2021 ^2^
Sapienic acid	(C16:1, *n*-10) MUFA	Component of sebum	Knox and Boyle, 2021 ^2^
Oleic acid	(C18:1, *n*-9) MUFA	Barrier function/regulating inflammation	Downing et al., 1986;^116^ Knox and Boyle, 2021 ^2^
Linoleic acid	(C18:2, *n*-6) PUFA	Barrier function/regulating inflammation	Ziboh et al., 2000;^22^ Knox and Boyle, 2021 ^2^
Arachidonic acid	(C20:4, *n*-6) PUFA	Proinflammatory properties	Calder, 2006;^25^ Knox and Boyle, 2021 ^2^
Eicosapentaenoic acid	(C20:5, *n*-3) PUFA	Proinflammatory properties	Knox and Boyle, 2021 ^2^
Docosahexaenoic acid	(C22:6, *n*-3) PUFA	Regulating inflammation	Knox and Boyle, 2021 ^2^

## 5 Hydrolipidic Mantle

The hydrolipidic mantle (HM) is a protective layer of the skin composed of a mixture of water and lipids, a natural emulsion formed by the products secreted by the sebaceous glands (lipids) plus epidermal lipids—emulsifiers— and sweat glands (water), forming a protective film and hindering transepidermal water loss.^37^ Its main function is to act as a barrier against external agents, maintaining hydration and protecting the skin against infections and irritations. The integrity of the hydrolipidic mantle is essential for skin health, as any imbalance can lead to dermatological problems such as dermatitis, acne, and premature aging, essentially because the skin becomes dry, and then the disease follows.^3^ Therefore, understanding and maintaining this natural barrier can lead to significant advances in the prevention and treatment of various esthetic conditions.^38^

The composition of the hydrolipidic mantle (Figure 5) is complex and varies between different areas of the body and among individuals. Factors such as age, race, sex, diet, and skincare habits can influence its composition and function. The application of topical products, such as moisturizers and sunscreens, can affect the composition of the hydrolipidic mantle, highlighting the importance of selecting appropriate products to maintain its integrity.^39^

**Figure 5:**
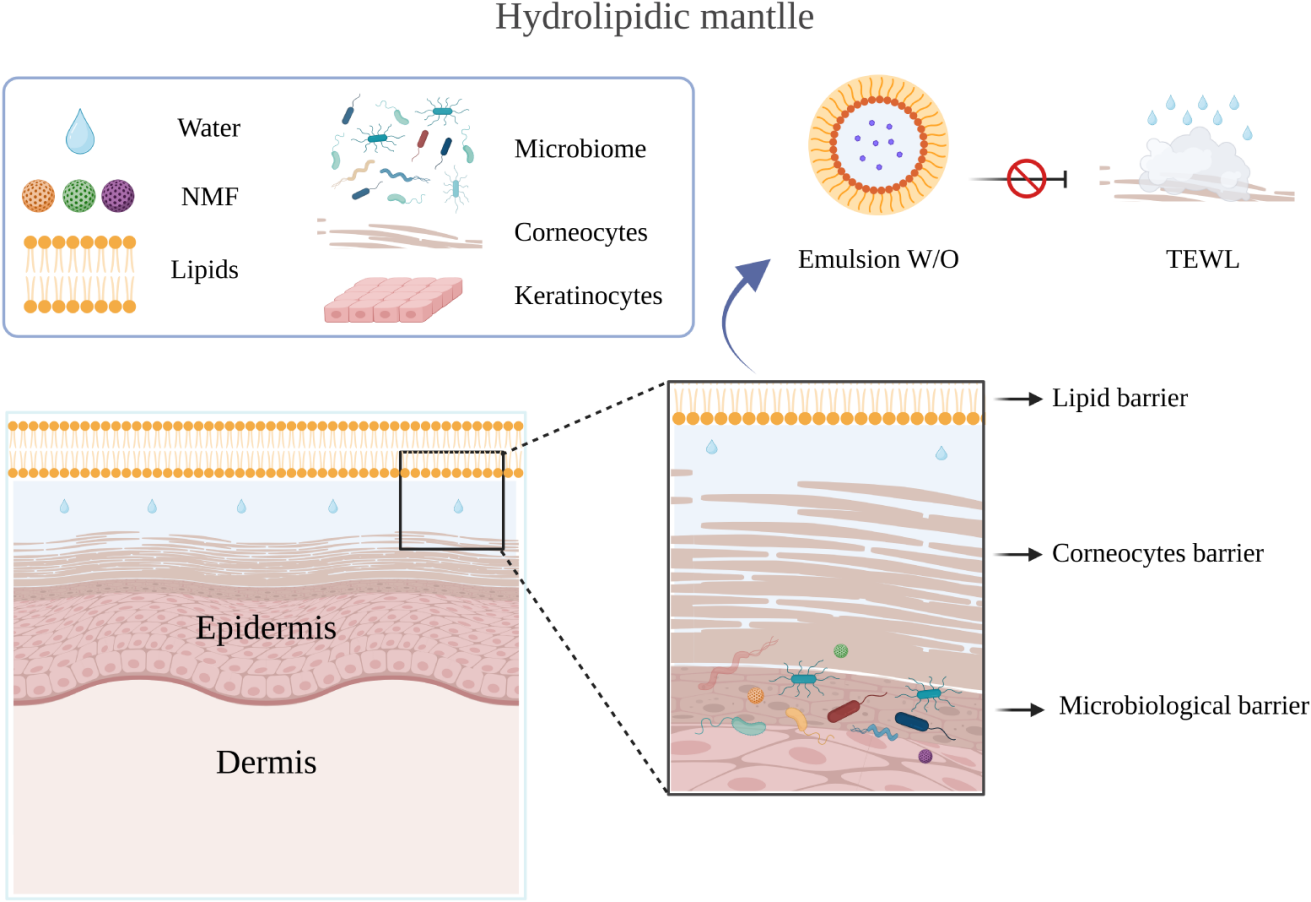
Hydrolipidic mantle and its main composition. Created with BioRender.com.

The intercellular lipid matrix, which represents 11% of the stratum corneum, is composed of ceramides, fatty acids, cholesterol, glycosylceramides, cholesterol sulfate, and phospholipids (which bind corneocytes together, forming a barrier to the passage of water).^37^ The composition of HM represents:

• 99% water;

• NMF—Natural Moisturizing Factor—17 amino acids: alanine, asparagine, citrulline, glycine, ornithine, proline, serine, among others; urocanic acid, pyrrolidone carboxylic acid (PCA), urea, lactic acid, lactate, potassium, chloride, sodium, phosphate, and citrate;

• Lipid matrix: 50% ceramides, 25% cholesterol, and 25% free fatty acids;

• Skin microbiota.

The most abundant substance found in HM is water (approximately 99%), which comes from the eccrine secretion of sweat glands. Water is responsible for maintaining the hydration of the stratum corneum.^40^ The natural moisturizing factor (NMF), which originates from the decomposition of keratinocytes and the union of the hydrolipidic mantle, is present on the surface of the skin and is responsible for maintaining the integrity of the epidermis, forming a chemical barrier of protection against aggressions from the external environment, maintaining the skin surface moisture, exhibiting antimicrobial activity, and reducing transepidermal water loss (TEWL).^37,40,41^

The lipid matrix is located on top of the water and provides protection against dehydration. In this region, we find two types of lipids: epidermal and sebaceous lipids, which are produced by the sebaceous gland and function as a natural emollient for the skin.^39^ Furthermore, they have an antimicrobial function, and the resident skin microbiota forms a biofilm, protecting the skin against the growth of pathogenic microorganisms.^42^

The skin’s function is to form a physical, chemical, and microbiological barrier, and its balance depends on preserving this barrier.^43,44^ The skin barrier is important not only for preventing substances from easily entering the tissues but also for preventing water loss and, consequently, dehydration.^40,44^ In addition, loss of the barrier function can lead to pH imbalances, inflammation, and skin dysbiosis.^44,45^

Almost a century ago, in 1928, the term “acid mantle” was first described by Schade and Marchionini as a metaphor for the low pH value of the skin.^44,46^ Years later, the same group described studies that showed the importance of pH value in maintaining the balance of the microbiota and skin homeostasis.^41,47^ Ali and collaboratorsdescribed the composition of free fatty acids from sebaceous lipids, microbiota metabolites, lactic acid, filaggrin catabolism products, sodium–hydrogen antiporter membrane type 1, and fatty acids from phospholipid hydrolysis as factors responsible for acidifying the skin’s pH value.^42,44^

Fatty acids help maintain the skin’s pH between 4 and 6.5, and pH controls physical properties and provides stability to membrane lipids by regulating the membrane structure under healthy conditions.^15^ In this sense, pH control is an important key to maintaining skin balance.^42^ Skin pH can vary due to a series of endogenous factors, such as age, genetics, anatomical region, sebum production, and pH imbalances are related to some pathologies such as atopic dermatitis, contact dermatitis, acne vulgaris, *Candida albicans* infection.^41^

A study conducted by Bouwstra and collaborators showed that the formation of lamellar structures in horny lipid mixtures was possible only at a low pH value. The formation of the stratum corneum depends on certain enzymes that are pH dependent; thus the pH of the skin is crucial for enzyme activity.^48^

## 6 Interaction between Microbiome and Lipids

The skin microbiota is composed of a variety of microorganisms, including bacteria, fungi, and viruses.^49^ These microorganisms coexist in a generally beneficial relationship with the host, contributing to immune defense and protection against pathogens.^6^ This microbiota begins to colonize the newborn’s skin during the birth process and can change throughout the life.^50,58^ By adulthood, the skin microbiota reaches a unique balance for each individual.^51^ The composition of the human skin microbiota is influenced by multiple factors, such as gender, environment, lifestyle, and hygiene practices.^52^ The most common bacterial species include *Staphylococcus epidermidis*, *Propionibacterium acnes*, and *Corynebacterium* spp.^53^ The correlation between skin lipids and skin microbiota is essential for maintaining skin health.

Lipids not only form a physical barrier but also modulate the composition and activity of the microbiota, contributing to immune defense and infection prevention. Skin lipids directly influence the composition and activity of the skin microbiota. Certain lipids, such as free fatty acids, have natural antimicrobial properties, inhibiting the growth of potentially harmful pathogens. In addition, the structure and organization of lipids in the *stratum corneum* can create a physical environment that favors the colonization of beneficial microorganisms.^53,54^

Free fatty acids present in the skin, such as oleic and linoleic acids, have been shown to possess significant antimicrobial activities. They act by destabilizing the cell membranes of microorganisms, leading to cell lysis. This mechanism is especially important in the regulation of pathogenic bacteria, helping to maintain the balance of the skin microbiota.^55^ The presence of ceramides in the skin can also influence the composition of the microbiota, favoring the colonization by beneficial microorganisms and inhibiting the proliferation of pathogens.^5^

Cholesterol, another important component of skin lipids, contributes to the stability of cell membranes and the formation of lipid lamellae. It can also influence skin microbiota by providing a substrate for certain microorganisms. One study suggests that cholesterol and its derivatives may have direct antimicrobial effects, although this area of research is still developing.^53^

Interventions that modify the lipid composition of the skin, such as the use of moisturizers containing ceramides or essential fatty acids, can help restore the balance of the skin microbiota. These products can improve barrier function and provide substrates necessary for the colonization of beneficial microorganisms, thereby promoting skin health.^3^

In murine models, *S. epidermidis* produces a sphingomyelinase that facilitates the acquisition of essential nutrients and concurrently enhances host ceramide synthesis, significantly increasing cutaneous ceramide concentrations and reducing water loss from compromised skin.^60^

Changes in the lipid composition of the skin can lead to imbalances in the skin microbiota, resulting in dermatological conditions, such as acne,^56^ atopic dermatitis, psoriasis,^57,58^ and even rosacea.^59^ For instance, a reduction in free fatty acid levels can allow the proliferation of pathogenic bacteria such as *Staphylococcus aureus*, while an excessive increase in sebum can favor the growth of *Cutibacterium acnes*.^60^ Furthermore, patients with atopic dermatitis may present increased pH values (5.7–6.2), low hydration, and an abundance of the pathogenic bacteria *Staphylococcus aureus*, which contribute to the pathophysiology of the disease.^44^

The microbiota interact with sebum lipids, such as triglycerides, breaking them down into free fatty acids and can also secrete certain lipids in a bidirectional manner. For instance, *Cutibacterium acnes* has the ability to secrete propionicin to defend against Gram-positive and Gram-negative anaerobic bacteria.^61,62^ The species *C. avidum* produces FFAs to acidify the skin and inhibit colonization by other pathogenic microbes (*S. aureus* and *Streptococcus pyogenes*).^61,63^ The species *Corynebacterium accolens* produces FFAs to inhibit *S. pneumoniae*.^59,61^ Already the *S. epidermidis* secretes 6-HAP or SCFAs to inhibit the growth of GAS, MRSA, *S. aureus*, and *C. acnes*.^64–68^

The growing understanding of the interaction between skin lipids and microbiota offers new opportunities for the development of targeted therapies. Products that combine probiotics and prebiotics with specific lipids may be a promising approach to treating skin disorders.^69^ Furthermore, personalizing skincare based on individual lipid composition and microbiota may lead to more effective and personalized treatments.^69,70^ Probiotics help restore the balance of the gut microbiota, thereby reducing systemic inflammation.^71^ Clinical studies have shown that supplementation with certain probiotics can improve skin conditions such as acne and atopic dermatitis by modulating immune pathways and improving barrier function.^72,73^

A study conducted by Buhas et al. in patients with psoriasis showed that oral supplementation for 12 weeks with probiotics (*Bacillus indicus* (HU36), *Bacillus subtilis* (HU58), *Bacillus coagulans* (SC208), *Bacillus licheniformis* (SL307), and *Bacillus clausii* (SC109)) and precision prebiotics (fructooligosaccharides, xylooligosaccharides, and galactooligosaccharides) resulted in changes in the intestinal microbiota and also in the profile of anti-inflammatory markers.^74,75^

The concept of the gut–skin axis suggests a complex bidirectional interaction between the gastrointestinal tract and the skin, mediated by immunological, hormonal, and microbial factors.^75^ Gut health can influence skin health, and vice versa.^76^

Gut dysbiosis, or imbalance in the microbiota, has been associated with several inflammatory conditions, including skin diseases.^77^ ^,^ Communication between the gut and the skin occurs through immunological and metabolic mechanisms.^7^ Cytokines and other inflammatory mediators produced in the gut can affect systemic inflammation and, consequently, the skin. Furthermore, metabolites produced by the gut microbiota, such as short-chain fatty acids (SCFAs), also called postbiotics can influence skin barrier function and lipid production in the skin.^78^

In recent years, there has been a significant increase in studies on the use of postbiotics, which are products obtained from the fermentation of microorganisms, and they have interesting properties to be explored in dermatological treatments, such as antioxidant, anti-inflammatory, and immunomodulatory.^79^ The use of butyrate, a SCFA, as a postbiotic in dermatology has also been reported. A study showed the use of butyrate in the treatment of psoriasis, presenting a response in defective Treg cells, in atopic dermatitis with an immunomodulatory effect, in the treatment of ulcers and also in protecting the skin against UVB radiation, exerting a significant reduction in the level of proinflammatory cytokines.^80^ This last approach is also interesting in cosmetic formulations and in the development of new products for skin treatment.

Another recent study evaluated an antiacne lotion containing yeast lysate produced by *Lactiplantibacillus plantarum* VHProbi E15 was applied to individuals with mild to moderate acne over 4 weeks.^81^ The results showed that the application of the topical antiacne lotion was safe and conferred numerous benefits to people with mild to moderate acne, the main ones being: significant improvement in lesions (*p* < 0.01) and reduction in sebum production and transepidermal water loss (*p* < 0.05), representing a promising therapeutic option for the treatment of acne.^81^

Diet and gut microbiota composition play critical roles in modulating skin barrier function and preventing inflammation. Dietary interventions that promote a healthy balance of gut microbiota, such as the inclusion of prebiotics, probiotics, and essential fatty acids, show promise^82^ and may improve skin health and treat dermatological conditions.

## 7 Diet and Skin Fatty Acids

Boelsma and collaborators have shown a significant correlation between diet and skin lipid composition, suggesting that nutrition may directly influence skin health. Diet is a key factor that may influence skin lipid composition.^83^ Essential nutrients, such as essential fatty acids (EFAs), vitamins, and antioxidants, play important roles in the synthesis and metabolism of skin lipids. A deficiency or excess of these nutrients may affect the production and function of skin lipids, impacting the integrity of the skin barrier.^20,83^

Essential fatty acids, such as linoleic acid (omega-6) and alpha-linolenic acid (omega-3), are critical dietary components that the human body cannot synthesize. These fatty acids must be obtained through the diet and are essential for the synthesis of ceramides and other skin lipids. Diets rich in essential fatty acids improve skin hydration and barrier function,^22^ and supplementation with specific lipids, such as ceramides and essential fatty acids, can significantly improve skin hydration and barrier function.^84^ However, a higher concentration of linoleic acid increases inflammation, so it must be dosed.

Dietary supplementation with omega-3 and omega-6 fatty acids has been shown to have significant benefits for skin health. For example, the intake of fish oil, which is rich in omega-3 fatty acids, has been associated with reduced inflammation and improved skin hydration. Additionally, borage oil, a rich source of gamma-linolenic acid (GLA), an omega-6 fatty acid, has shown efficacy in treating conditions such as atopic dermatitis.^3^

Diets rich in saturated fatty acids and low in polyunsaturated fatty acids have been associated with increased skin inflammation.^86^ Conversely, diets rich in omega-3 fatty acids can reduce inflammation and improve conditions such as psoriasis and atopic dermatitis.^85^

Recent studies have shown that diet establishes a direct relationship with specific biochemical markers and the transcription of genes related to the function of the sebaceous glands, as well as the proliferation of bacteria and inflammation that stimulate the progression of acne vulgaris.^43^

Vitamins such as vitamins E and C are important antioxidants that protect skin lipids against free-radical-induced lipid peroxidation. Vitamin E is incorporated into lipid membranes, where it acts as a defense against oxidative stress. Antioxidant deficiency can lead to lipid damage and skin barrier dysfunction, highlighting the importance of an antioxidant-rich diet for skin health.^22^ Complementary essential fatty acid supplementation has been used in the management of atopic dermatitis, while anti-inflammatory diets have been shown to be beneficial in psoriasis. Similarly, diets rich in fruits, vegetables, and fiber are associated with improved skin barrier function and reduced incidence of inflammatory skin conditions.^88^ These approaches highlight the importance of considering diet as an integral part of therapeutic strategies for skin conditions.^87^

## 8 Correlation Lipids and Aging

Skin aging is a complex process influenced by intrinsic and extrinsic factors, including genetics, sun exposure, pollution, and diet.^89^ Fatty acids, essential components of cell membranes and skin lipids, play a crucial role in maintaining skin integrity and preventing premature aging.

Aging is associated with significant changes in the lipid composition of the skin. Studies show that the production of ceramides, cholesterol, and free fatty acids decreases with age, resulting in a less effective skin barrier. This change contributes to increased dryness, sensitivity, and susceptibility to skin diseases in older adults.^18^ With aging and the effects of lipid decrease, the skin can present a weakened skin barrier and increased TEWL—transepidermal water loss.^89^ This contributes to dry skin, wrinkling, and loss of elasticity. Boelsma and collaborators show that supplementation with essential fatty acids can help restore barrier function and improve hydration and the appearance of aging skin.^20^

Sebum also plays an important role in moisturizing and protecting the skin. However, overproduction of sebum, which is common in oily skin, can have negative long-term effects. Excess sebum can oxidize on the skin’s surface, generating free radicals that damage skin cells and contribute to premature aging.^14^ In addition, oily skin is more susceptible to the formation of enlarged pores and irregular texture, characteristics that can be exacerbated over the years.

With aging and changes in the sebaceous gland activity, imbalances in the skin microbiome can occur. Studies indicate that the skin microbiome, including *C. acnes*, influences the skin aging process.^90^ These changes contribute to the degradation of the extracellular matrix and the loss of skin elasticity, accelerating the appearance of wrinkles and other signs of aging.

Vitamin E or α-tocopherol, a fat-soluble vitamin present in sebaceous secretion, has been helping to protect the skin from free radical damage. These free radicals, generated by exposomes such as UV exposure and environmental pollutants, contribute significantly to skin aging by damaging dermal collagen and elastin. Supplementation with α-tocopherol may provide additional antioxidant protection, delaying signs of aging.^22^

Chronic low-grade inflammation is an important factor in skin aging and can lead to the degradation of collagen and elastin, which are proteins essential for skin firmness and elasticity. For example, the presence of *C. acnes* in oily skin is often associated with chronic inflammatory states, which can accelerate the aging process.^91^ However, with aging, the microbial diversity could increase, although some more abundant microorganisms of the skin tends to decrease like *Lactobacillus* and *Cutibacterium*.^92–94^ *C. acnes* may become less dominant in older individuals, which can affect skin barrier function and local immune response.^95^ These changes may contribute to an increased susceptibility to skin infections and decreased skin quality over time.

Rogers et al. found a significant decline in all major lipid species, particularly ceramides, with increasing age. Likewise, lipid levels in the stratum corneum across all examined body sites were markedly reduced during the winter compared to spring and summer. Notably, relative levels of ceramides with linoleic acid were diminished both in winter and in aged skin, while ceramides with oleic acid levels showed an increase under the same conditions.^96,98^

Clinical studies have explored the efficacy of fatty acid supplements in promoting the health of aging skin. Results indicate that regular supplementation may improve skin elasticity, reduce wrinkles, and increase hydration. Furthermore, essential fatty acids have shown the potential to accelerate wound healing and improve skin quality in elderly patients.^87^

## 9 Cosmetic Formulations Based on Skin Lipids

The search for cosmetic products that promote skin health and beauty has driven the development of innovative formulations.^97^ The selection of products that balance the pH value and preserve the hydrolipidic mantle is necessary in dermatologists’ offices, and the use of products that allow a pH value of around 5.5 can help in the treatment of various skin conditions.^41^ Among the most promising ingredients, fatty acids stand out for their beneficial properties for the skin. Cosmetic formulations based on fatty acids offer multiple benefits for the health and appearance of the skin^98^ Essential fatty acids, in particular, play a crucial role in maintaining the integrity of the skin barrier, hydration, and modulation of inflammation. The inclusion of fatty acids in cosmetic products can significantly improve skin health, providing hydration, antioxidant protection, and anti-inflammatory properties.

Several studies have investigated the efficacy of cosmetic formulations based on fatty acids.^97^ Results indicate that products containing essential fatty acids can improve barrier function, increase hydration, reduce inflammation, and promote skin healing. These benefits make fatty acids valuable components in cosmetic products intended for skin care and rejuvenation.^3,97^

Environmental factors, such as climate, pollution, and the use of topical products, also affect the lipid composition of the skin. Products containing harsh surfactants or alcohol can extract essential lipids, exacerbating dryness and irritation of the skin. Therefore, choosing the right skincare products is essential to maintaining the integrity of the lipid barrier.^98^

Daily skincare has a significant impact on the relationship among *C. acnes*, oily skin, and aging. Oil-control products, such as cleansers and toners, can help regulate sebum production and reduce the proliferation of *C. acnes*.^99^ However, it is important to choose products that maintain the balance of the microbiome, avoiding excessive natural lipids, which can compromise the skin barrier and accelerate aging. Future studies should focus on optimizing the stability of formulations, exploring new sources of fatty acids, and investigating the molecular mechanisms through which they exert their beneficial effects on the skin.

The fatty acids used in cosmetic formulations can be classified as saturated, monounsaturated (MUFA), and polyunsaturated (PUFA) fatty acids. Essential fatty acids, such as linoleic acid (omega-6) and alpha-linolenic acid (omega-3), are often incorporated into products due to their beneficial properties. Additionally, fatty acids such as oleic acid (omega-9) and lauric acid are used for their emollient and antimicrobial properties.^53^ Fatty acids also possess anti-inflammatory properties that can reduce skin irritation and inflammation, promoting healthier and more balanced skin.^4^

Kim et al. utilizing Raman spectroscopy demonstrated that ceramide-containing cosmetic formulations exert a beneficial effect on skin absorption, as evidenced by both visual and statistical outcome analyses. Accordingly, ceramides, when appropriately formulated, should be considered critical constituents in dermatological formulations intended to support the maintenance and restoration of the skin’s primary function as a permeable barrier.^87^

Despite their benefits, incorporating fatty acids into cosmetic formulations poses several challenges. Polyunsaturated fatty acids are susceptible to oxidation, which can compromise product stability and efficacy. To overcome this problem, antioxidants such as vitamin E are often added to formulations to prevent fatty acid degradation.^99^

Moisturizing is one of the main functions of fatty acid-based cosmetic formulations. Creams and lotions containing essential fatty acids help to reinforce the skin barrier and maintain hydration, thereby improving the skin elasticity and texture. Studies show that products containing omega-3 and omega-6 fatty acids can significantly improve skin hydration and reduce TEWL.^20^

The anti-inflammatory properties of fatty acids are particularly useful in the treatment of inflammatory skin conditions, such as atopic dermatitis and psoriasis. Cosmetic formulations incorporating omega-3 and omega-6 fatty acids have been shown to reduce inflammation and improve the appearance of the skin in individuals with these conditions. These fatty acids help to modulate the inflammatory response and reduce the production of proinflammatory cytokines.^25^

## 10 Skin Care Routines

Surfactants are widely used in personal care products, such as shampoos, soaps, and toothpastes. Their popularity is due to their effectiveness as cleansing and foaming agents. However, the continued and indiscriminate use of sodium lauryl sulfate (SLS) and sodium lauryl ether sulfate (SLES) has raised concerns about their effects on the skin, especially in terms of irritation and impairment of the skin barrier.^99,101^ Sodium lauryl sulfate (SLS) is an anionic detergent known for its ability to remove oil and dirt from the skin and hair. Due to its chemical structure, SLS is highly effective in reducing the surface tension of water, allowing it to spread and penetrate surfaces more easily, which contributes to its cleansing action.^100,101^ SLS can compromise the skin barrier by removing the skin’s natural lipids, and this can result in transepidermal water loss, leaving the skin dry, irritated, more susceptible to damage^99^ and prone to the penetration of potentially irritating or allergenic substances.^53^

SLS can cause skin irritation, especially in individuals with sensitive skin.^97^ Irritation occurs because SLS can destabilize the cellular layers of the epidermis, causing inflammation and redness. This reaction is more evident at higher concentrations of SLS or with prolonged use, suggesting the need for balanced formulations that minimize this risk.^100,102^ A study by Effendy and Maibach found that, compared with other common surfactants, SLS caused a higher rate of erythema (redness of the skin) and dehydration. These results reinforce the need for caution when choosing products containing this ingredient, especially for people with sensitive skin.^103^

In response to concerns about SLS (anionic), many companies have sought gentler alternatives. Ingredients such as lauryl glucoside (neionic) and cocamidopropyl betaine (amphiphilic) are gaining popularity because they are less irritating and better preserve the skin’s lipid barrier. Furthermore, many brands are reformulating their products to include lower levels of SLS or combining it with moisturizing and emollient agents that counteract its potentially harmful effects.^104^ Currently, SLES is not used in the formulations; instead acyl glutamate is used.

Cosmetic treatment routines can directly influence the health and balance of the skin. For example, the use of sodium hydroxide-based soaps and cleansers can impact the skin in several ways. One of the main reasons is the high alkalinity of these products. Soaps with a high pH, typical of products containing sodium hydroxide residues, can destabilize the lipid barrier, resulting in dryness, irritation, and even inflammation of the skin.^105^ Studies show that regular use of highly alkaline soaps and cleansers can cause skin irritation, especially in people with sensitive skin or preexisting conditions such as atopic dermatitis. Residual sodium hydroxide in hygiene products can dehydrate the skin by removing the natural lipids that maintain skin hydration, making the skin more susceptible to cracking and irritation.^106^

Skincare has a significant impact on the relationship with oily skin. However, it is important to choose products that maintain the balance of the microbiome, avoiding excessive sebum shedding, which can compromise the skin barrier and accelerate aging. Often, people overcleanse to control shine and oiliness, completely removing the hydrolipidic mantle, making it unable to maintain surface moisture, leaving it unstructured, and with a strong tendency toward dryness. Because of this, cosmetics for mature skin have a higher degree of oiliness in their base formulation.^37^

## 11 Conclusion

In this study, we conducted a literature review of the key lipids of the skin, with a focus on fatty acids and their significance. Understanding the function and properties of these lipids is essential for maintaining healthy skin, preserving barrier function and supporting microbiome balance. Fatty acid-based formulations play a crucial role in restoring skin homeostasis. As future research opportunities, the application of multiomics technologies can be employed, such as metabolomics, in order to elucidate the role of fatty acids and their implications for skin health, correlating with dermatological diseases. Further research is needed to better understand the relationship between skin health and lipids, as well as the role of nutrition—an important epigenetic factor—and its influence on the skin’s sebaceous composition.
